# Supplementary material for: Comparison of balance changes after inspiratory muscle or Otago exercise training
Source: PLoS One. 2020 Jan 24;15(1):e0227379. doi: 10.1371/journal.pone.0227379 (PMC6980667; doi:10.1371/journal.pone.0227379)
Supplement: S1 Appendix — (DOCX) [file pone.0227379.s001.docx]

Appendix 1 – The Otago exercises program

The training program was provided following guidelines of the Geriatric Medicine Department, The Royal Bournemouth and Christchurch Hospital NHS Foundation Trust (Fairmile Road, Christchurch, UK)

| **Warm-up exercises.**  Always begin with a warm-up to prepare your body for the main exercises. | | |  |  |
| --- | --- | --- | --- | --- |
|  | - Chair March   - Sit tall on the front third of the chair seat, away from the chair back - Hold the sides of the chair - Alternately lift your feet and place them down with control - Build to a rhythm that is comfortable for you - Continue for 30 seconds. | |  |  |
|  | - Arm swings   - Sit tall away from the chair back - Place your feet flat on the floor below you knees - Bend your elbows and swing your arms from the shoulder - Build to a rhythm that is comfortable for you - Continue for 30 seconds. | |  |  |
|  | - Head movements   - Sit tall, away from the back of chair - Turn your head slowly to the left - Return to start position and turn to the right side - Repeat 5 times. | |  |  |
|  | - Neck movements   - Sit tall and place one hand on your chin - Slowly guide chin straight back with your hand (not bending the neck back or forwards) - Relax and repeat 5 times. | |  |  |
|  | - Back extension   - Sit tall, on front third of chair, way form back of chair - Place hands on bottom just below small of back - Lift chest and gently arch backward - Repeat 5 times. | |  |  |
|  | - Trunk movements   - Sit tall with your feet flat on the floor, hip-width apart - Fold your arms across your chest - Check your posture, with control, turn your upper body and head towards you left - Repeat on the opposite side - Repeat 5 times. | |  |  |
|  | - Ankle movements   - Sit tall away from the back of the chair - Hold the sides of the chair - Place the hell of one foot on the floor, then lift and put the toe down in the same spot - Repeat 5 times on each leg. | |  |  |
| 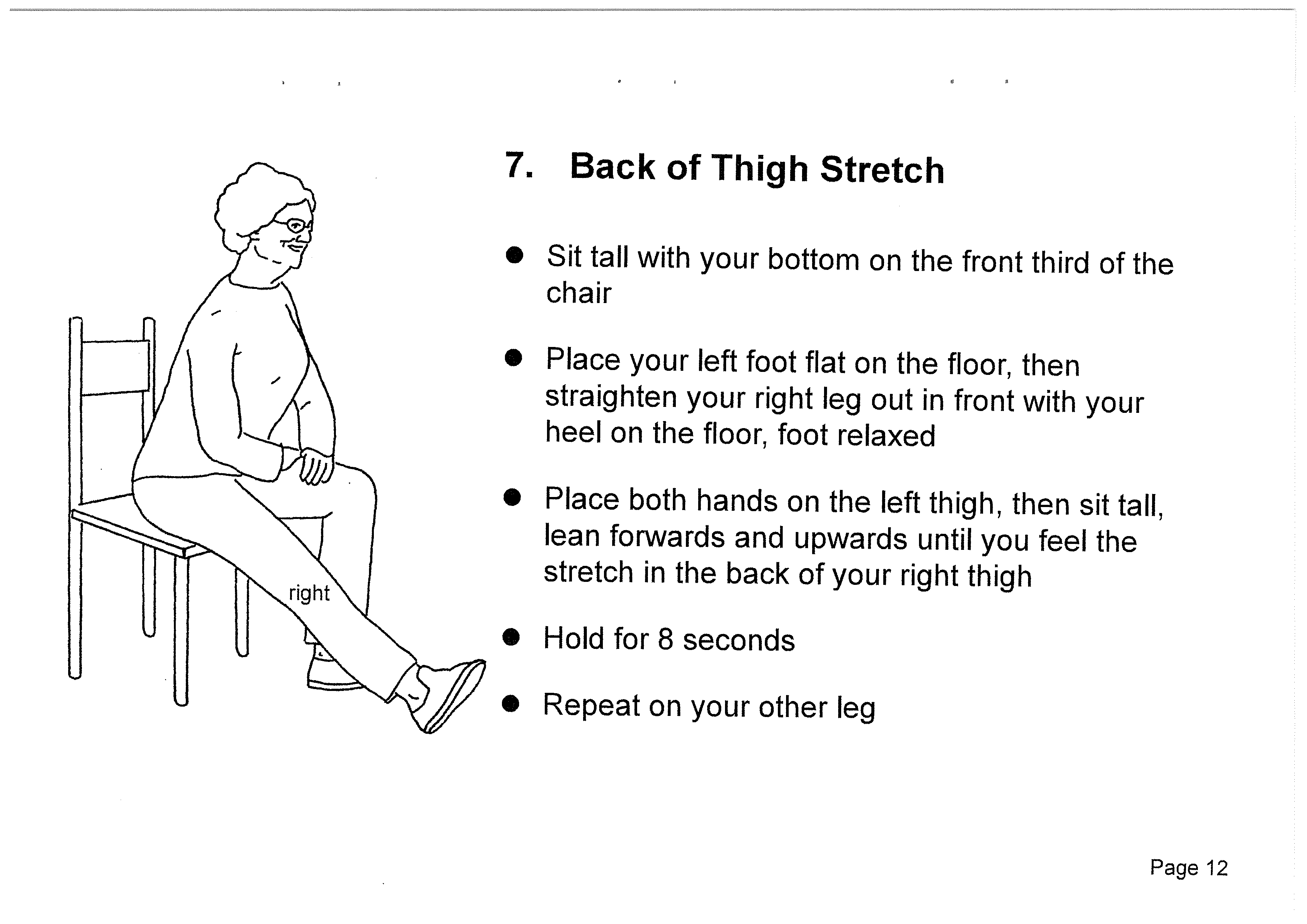 | - Back of thigh stretch   - Sit tall with your bottom on the front third of the chair - Place your left foot flat on the floor, then straighten your right leg out in front with your heel on the floor, foot relaxed - Place both hands on the left thigh, then sit tall, lean forward and upward until you feel the stretch in the back of your right thigh - Hold for 8 seconds - Repeat on your other leg. | |  |  |
| 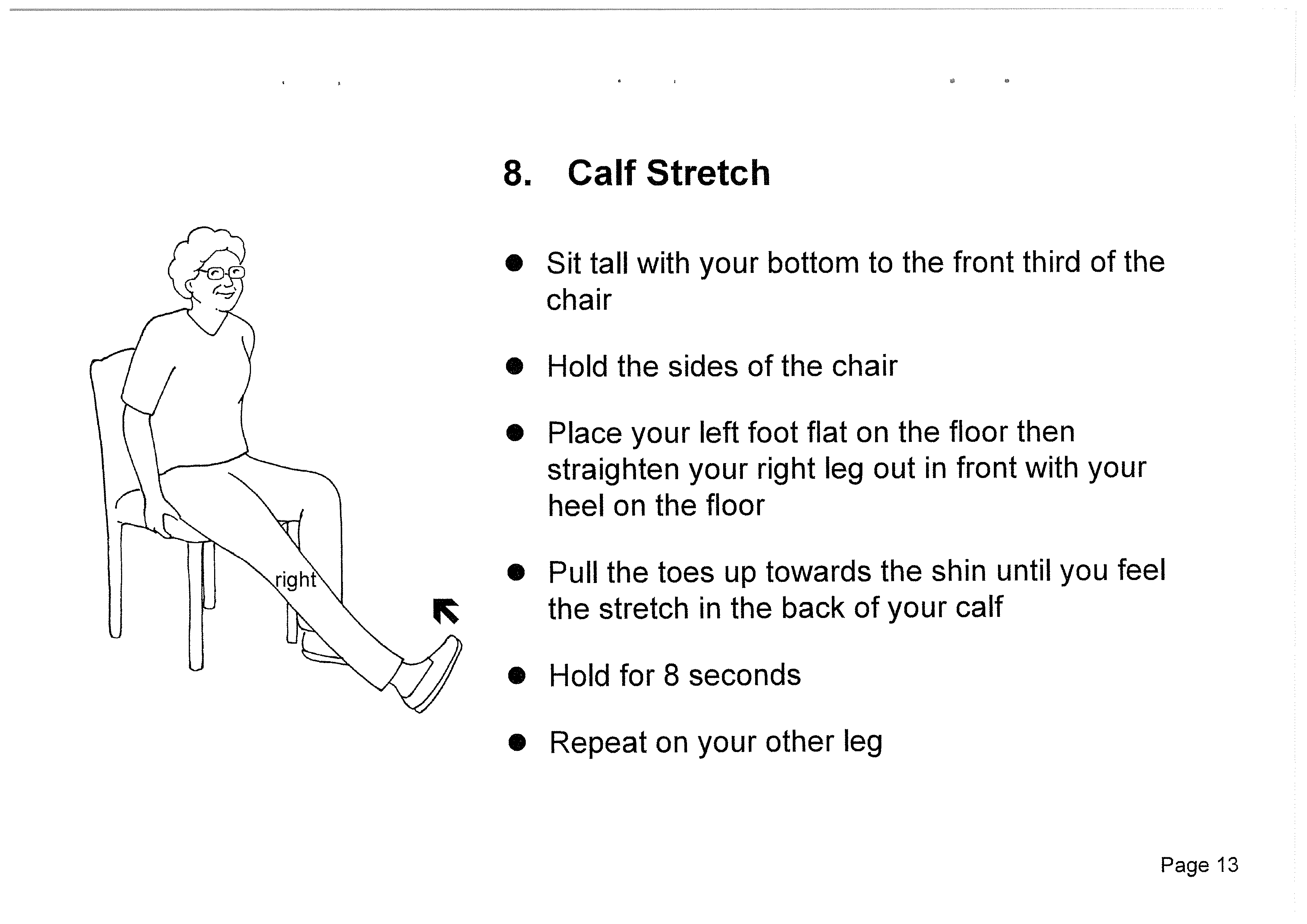 | - Calf Stretch   - Sit tall with your bottom to the front third of the chair - Hold the sides of the chair - Place your left foot flat on the floor then straighten your right leg out in front with your heel on the floor - Pull the toes up towards the shin until you feel the stretch in the back of your calf - Hold for 8 seconds - Repeat on your other leg. | |  |  |
| **Exercise to help improve your bone and muscle strength**  Build up to 10 repetitions of each exercise on each leg as you progress then build up to 2 or more sets of 10. | |  |  |  |
| 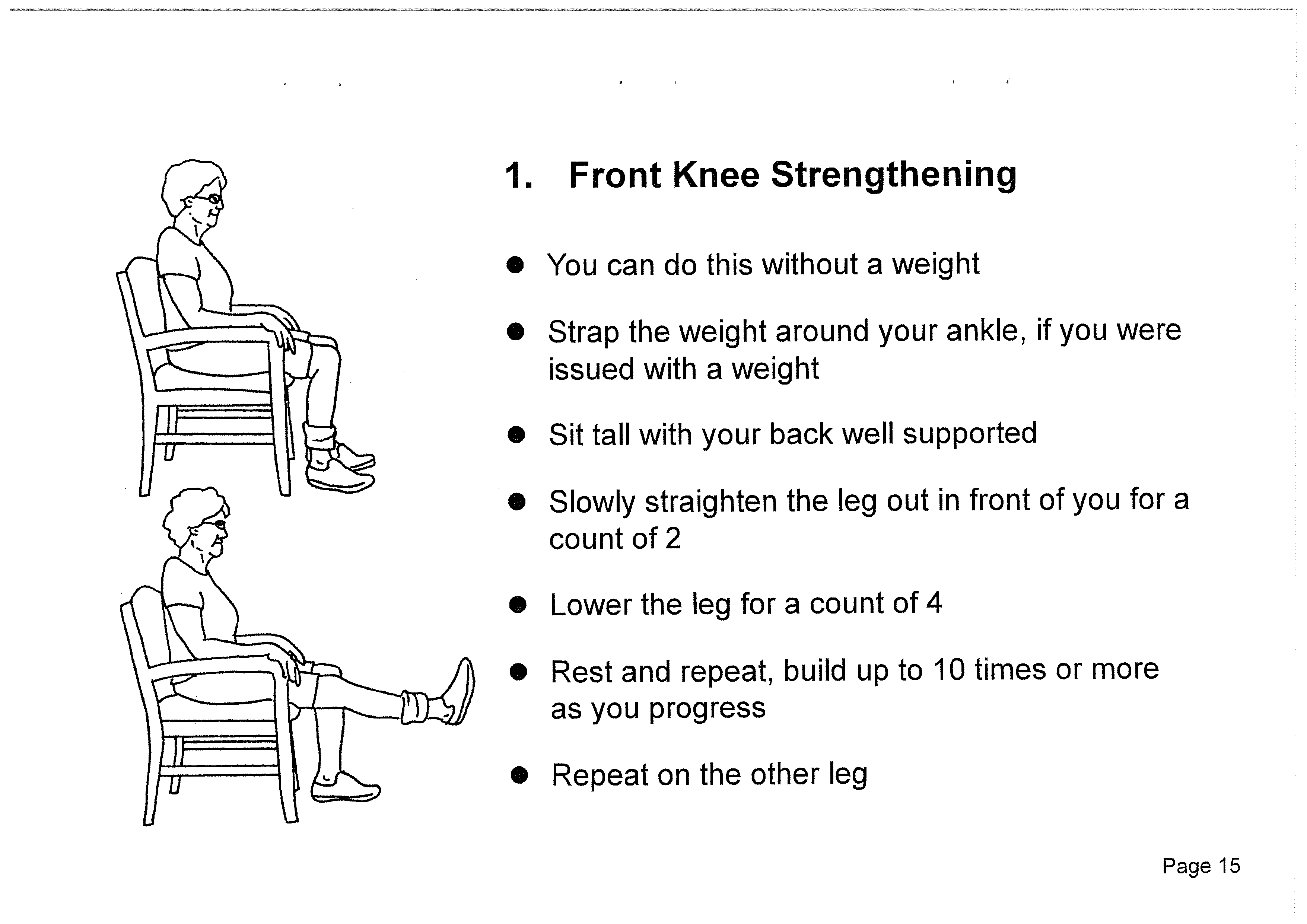 | - Front knee strengthening   - You can do this without a weight - Strap the weight around your ankle, if you were issued with a weight - Sit tall with your back well supported - Slowly straighten the leg out in front of you for a court of 2 - Lower the leg for a count of 4 - Rest and repeat, build up to 10 times or more as you progress - Repeat on the other leg. | |  |  |
| 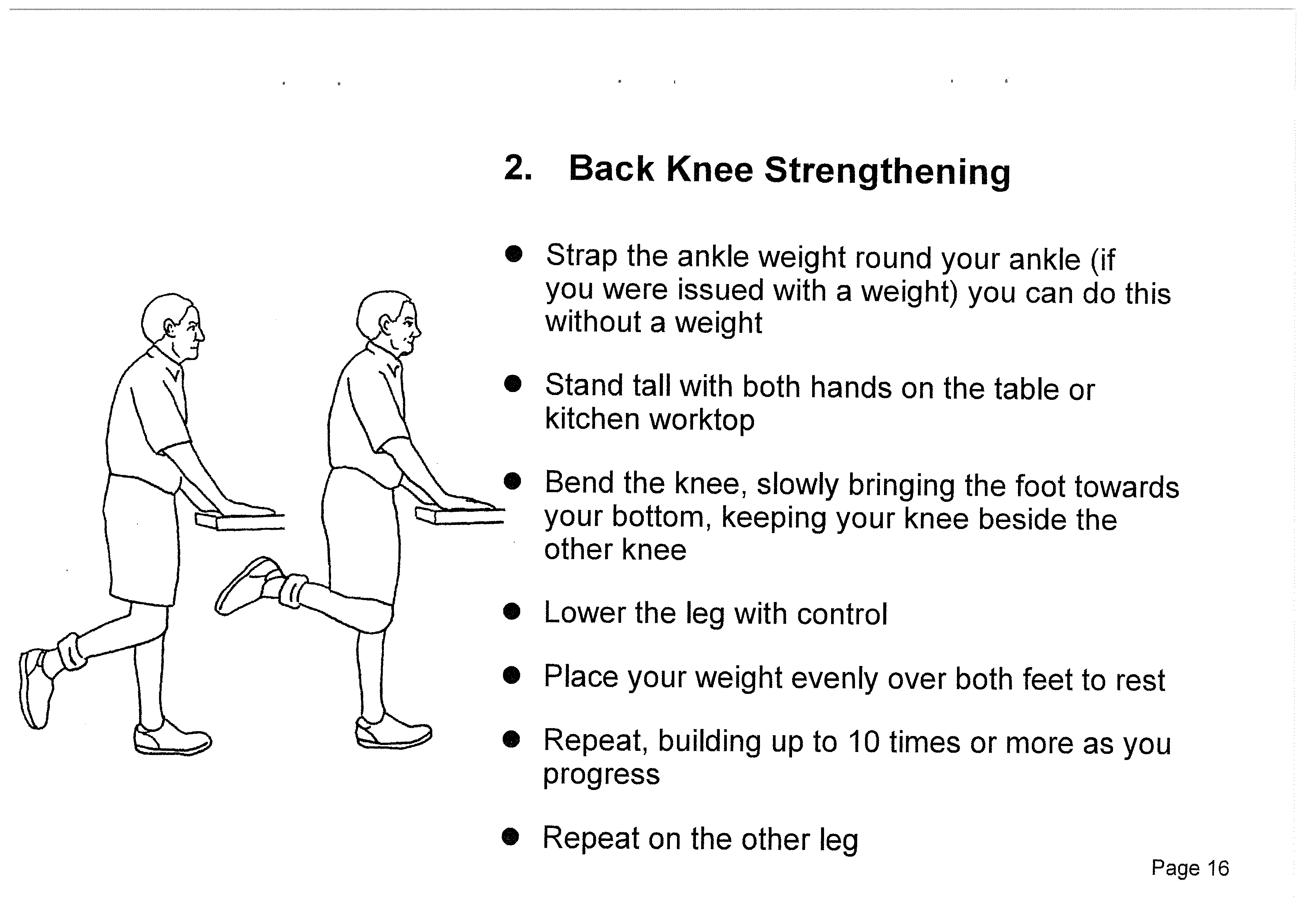 | - Back knee strengthening   - Strap the ankle weight around you ankle (if you were issued with a weight) you can do this without a weight - Stand tall with both hands on the table or kitchen worktop - Ben the knee, slowly bringing the foot towards your bottom, keeping your knee beside the other knee - Lower the leg with control - Place your weight evenly over both feet to rest - Repeat building up to 10 times or more as you progress - Repeat on the other leg. | |  |  |
| 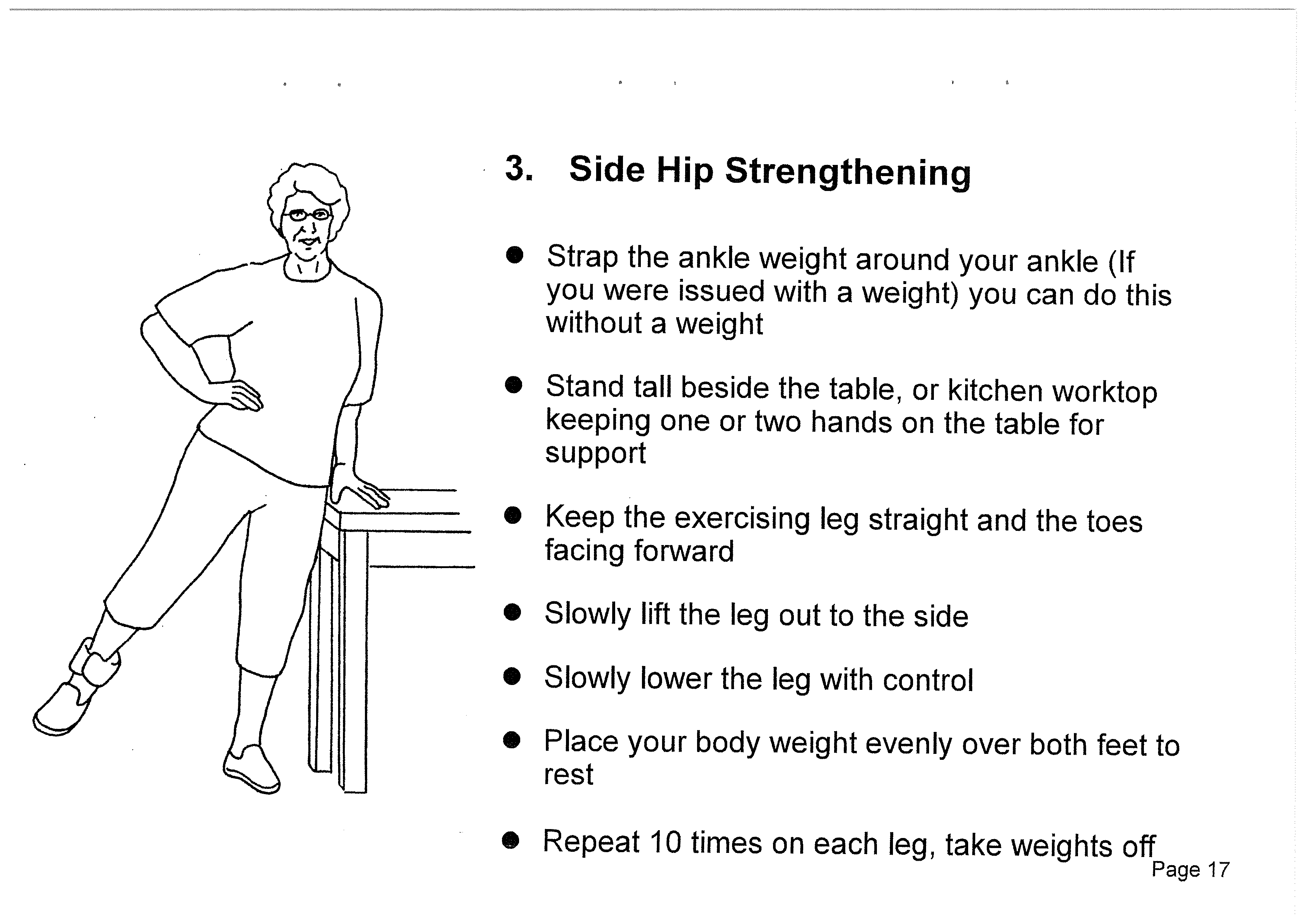 | - Side hip strengthening   - Strap the ankle weight around your ankle (if you were issued with a weight) you can do this without a weight - Stand tall beside the table, or kitchen worktop keeping one or two hands on the table for support - Keep the exercising leg straight and the toes facing forward - Slowly lift the leg out to the side - Slowly lower the leg with control - Place your body weight evenly over both feet to rest repeat 10 times on each leg take weights off. | |  |  |
| 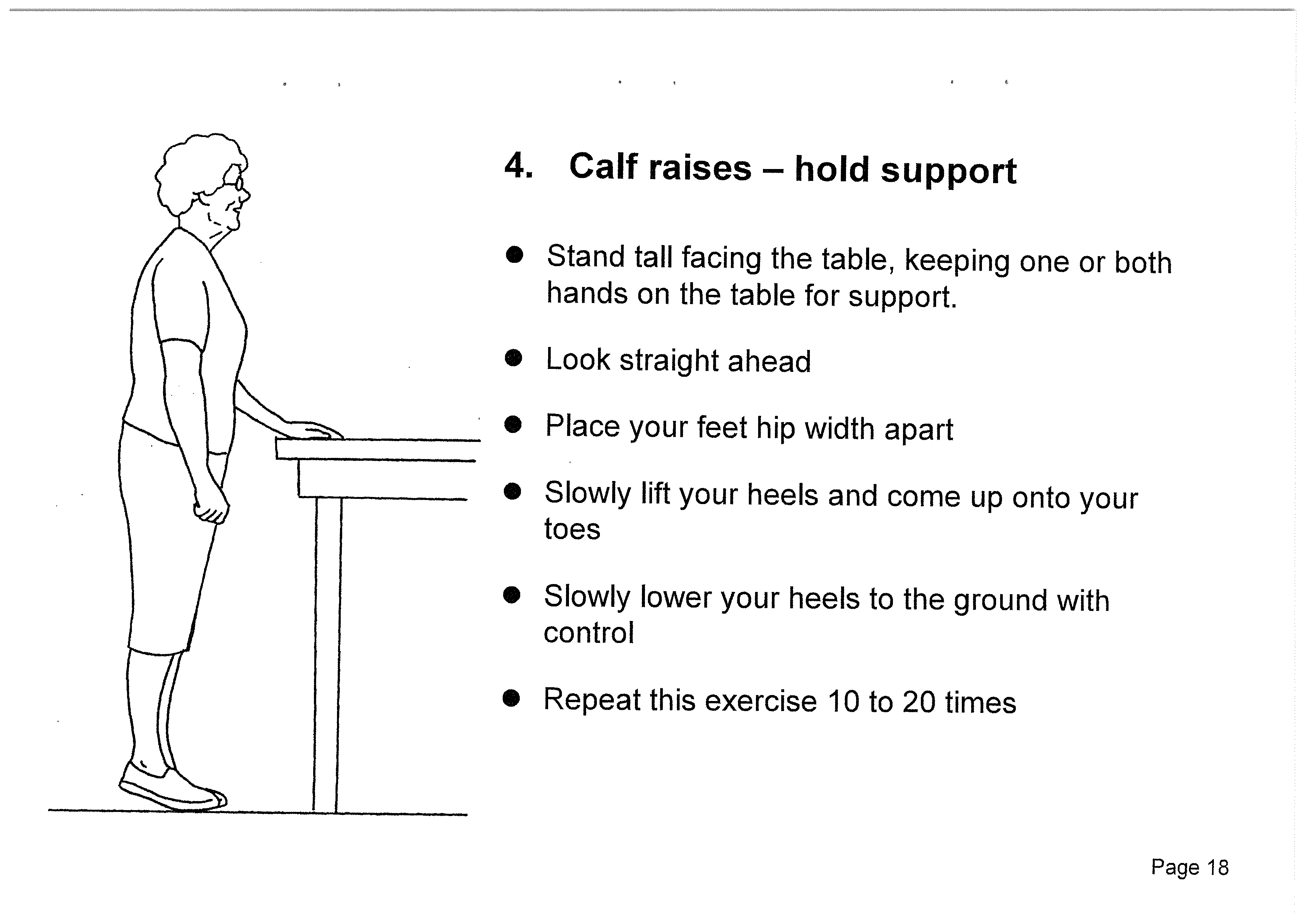 | - Calf raises   - Stand tall facing the table, keeping one or both hands on the table support - Look straight ahead - Place your feet hip-width apart - Slowly lift your heels and come up onto your toes - Slowly lower you heels to the ground with control - Repeat this exercises 10 to 20 times. | |  |  |
| 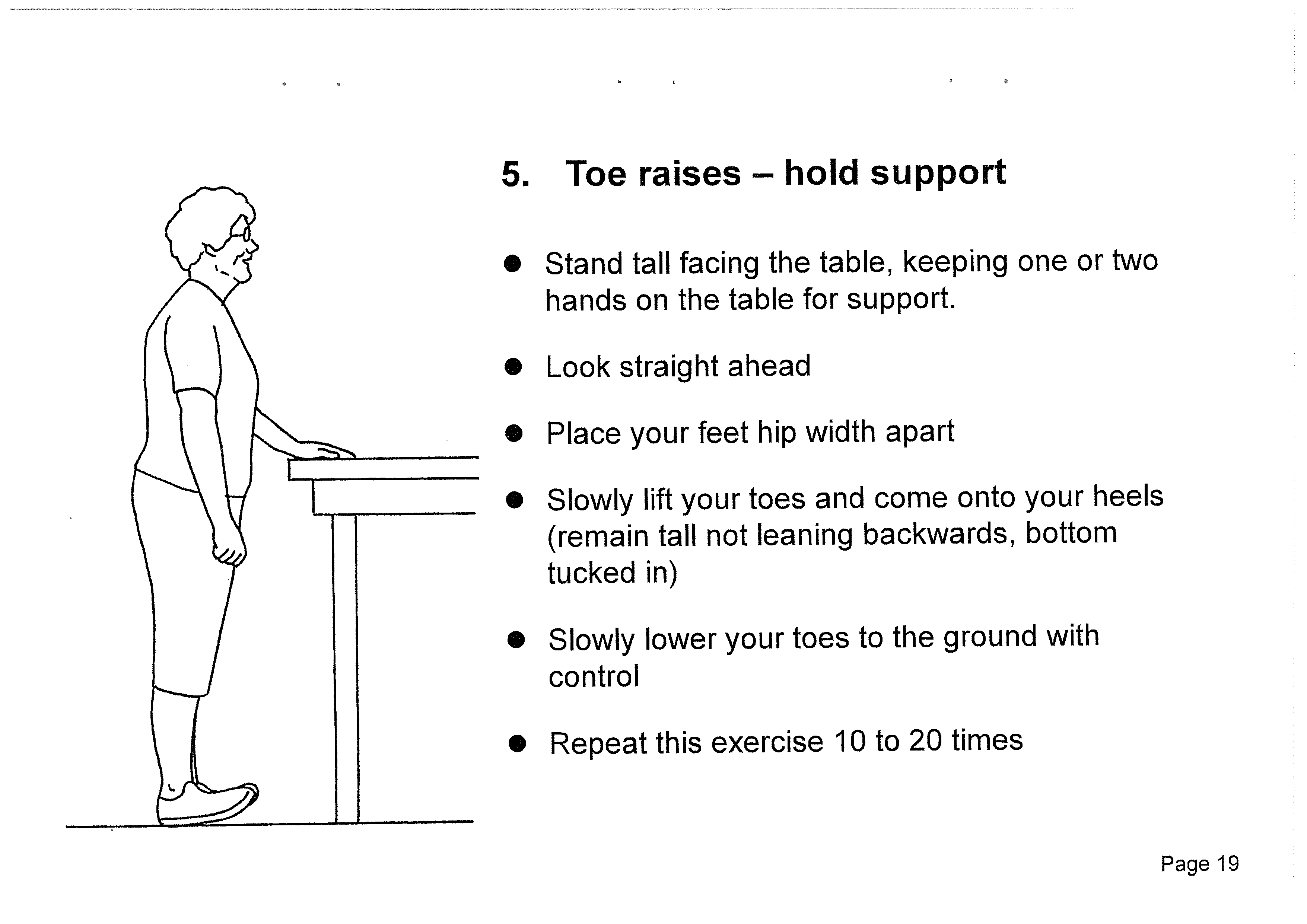 | - Toe raises   - Stand tall facing the table, keeping one or two hands on the table for support - Look straight ahead - Place your feet hip-width apart - Slowly lift your toes and come onto your heels (remain tall not leaning backwards, bottom tucked in) - Slowly lower your toes to the group with control - Repeat this exercises 10 to 20 times. | |  |  |
| **Exercises to help improve your balance**  Do these holding on with both hands for support and as you progress reduce the support to holding with one hand, support with fingertips etc. | |  |  |  |
|  | - Knee bends   - Stand tall with both hands on the table, feet hip-width apart - Take bottom backwards and bend knees as if to sit down, make sure heels don-t lift and knees are above toes - Slowly push through both feet to stand up again - Repeat 5 times initially and build up to 10 times. | |  |  |
|  | - Heel-toe standing   - Stand tall beside the table or worktop - Look straight ahead - Lift your heels and come up onto your toes - Slowly walk 10 steps on your toes - Bing the back foot beside the front foot and lower the heels to the ground - Turn around and walk 10 steps on your toes in the opposite direction. | |  |  |
|  | - Heel-toe standing   - Stand tall beside the table or worktop - Look straight ahead - Place one foot directly in front of the other foot so that the feet form a straight line - Hold this position for 10 seconds - Now bring the back foot directly in front of the other foot - Hold this position for 10 seconds. | |  |  |
|  | - Heel toes walking   - Stand tall beside the table or worktop - Look straight ahead - Place one foot directly in front of the other so that the feet form a straight line and repeat for 10 steps - Turn around and walk 10 steps in the opposite direction. | |  |  |
|  | - One leg stand   - Stand tall beside the table or worktop - Look straight ahead - Balance on one leg, keep the knees clos3e together - Hold this position for 10 seconds place the foot down and repeat on the other leg. | |  |  |
|  | - Side walk   - Stand tall holding the chair or kitchen worktop with both hands, feet hip-width apart (when confident, try holding with only one hand) - Take a step to the right and then to the left - Continue for 30 seconds - Now try to take 2 steps to the right and then two to the left continue for 30 seconds - Alternatively stand at kitchen surface and take 5 small steps to the side and repeat in opposite direction - Build up to 10 steps in each direction. | |  |  |
|  | - Heel walking   - Stand tall beside the table or worktop - Lift your toes and come onto your heels - Look straight ahead, bottom tucked in - Walk 10 steps forward on your heels - Bring the back foot beside the front foot and lower the toes to the ground with control - Turn around and walk 10 steps on your heels in the opposite direction. | |  |  |
| 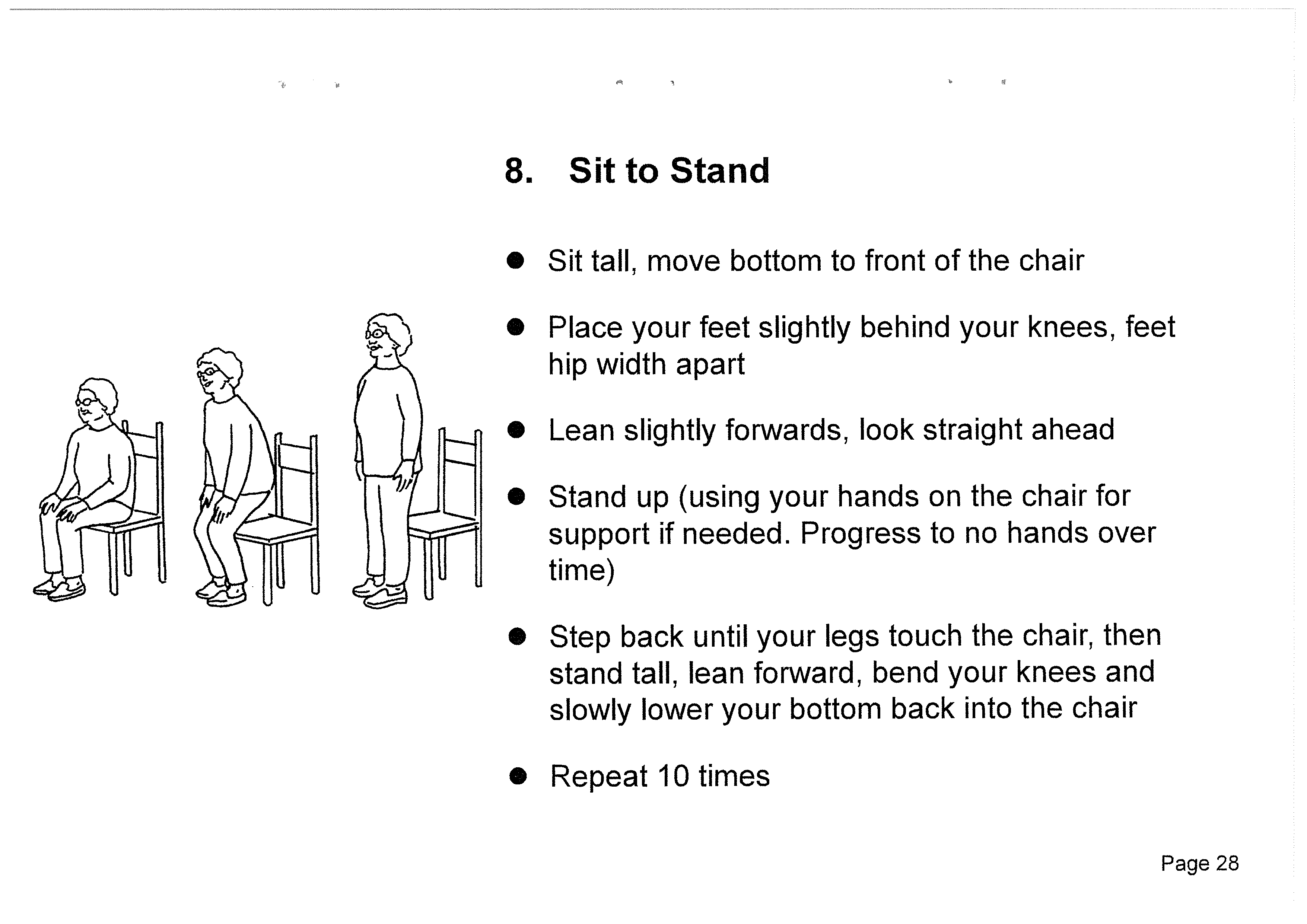 | - Sit to stand   - Sit tall, move bottom to front of the chair - Place your feet slightly behind your knees, feet hip-width apart - Lean slightly forwards, look straight ahead - Stand up (using your hands on the chair for support if needed. Progress to no hands over time) - Step back until your legs touch the chair then stand tall, lean forward bend your knees and slowly lower your bottom back into the chair - Repeat 10 times. | | | |
| 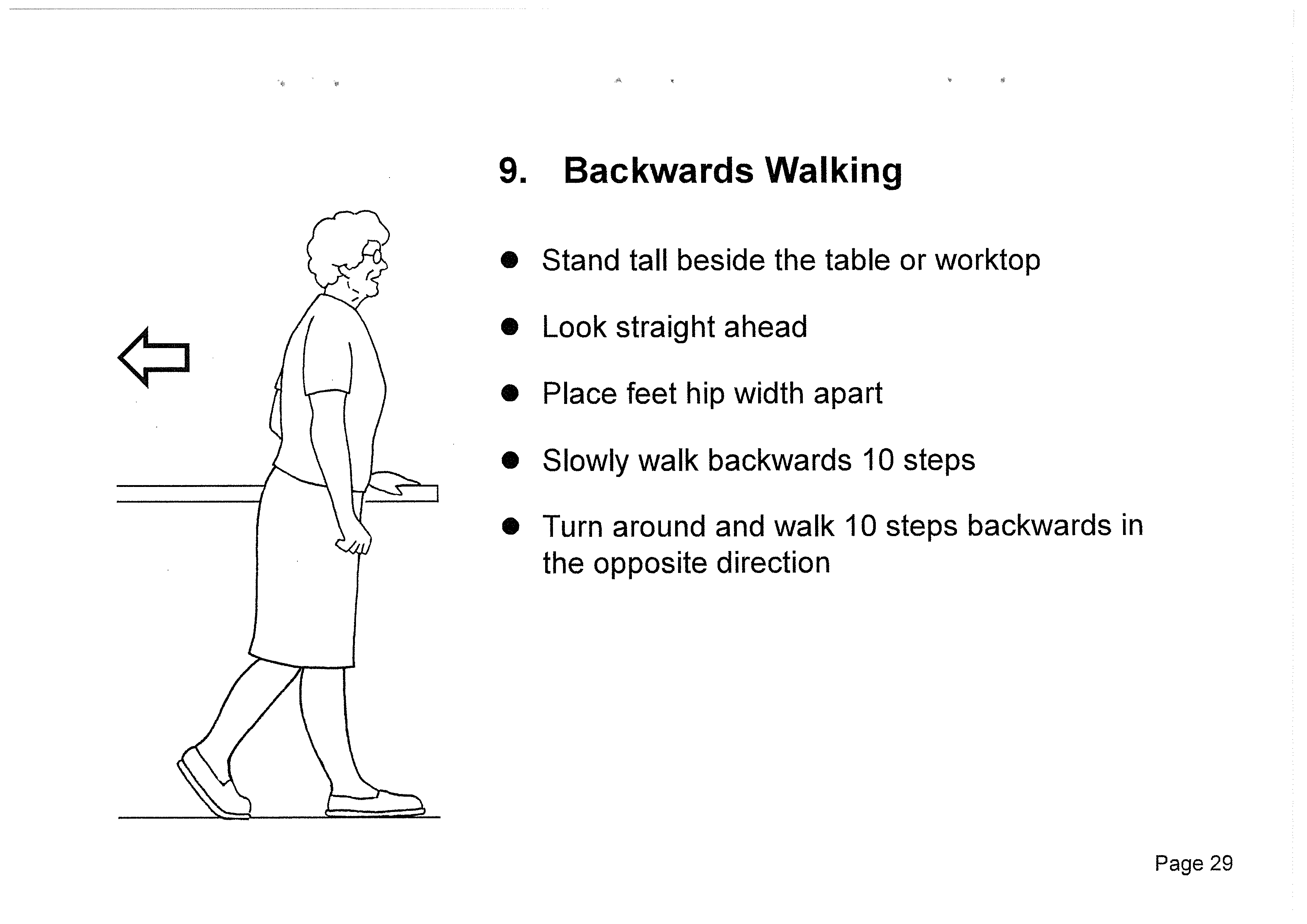 | - Backwards walking   - Stand tall beside the table or worktop - Look straight ahead - Place feet hip-width apart - Slowly walk backwards 10 steps - Turn around and walk 10 steps backwards in the opposite direction. | |  |  |
| 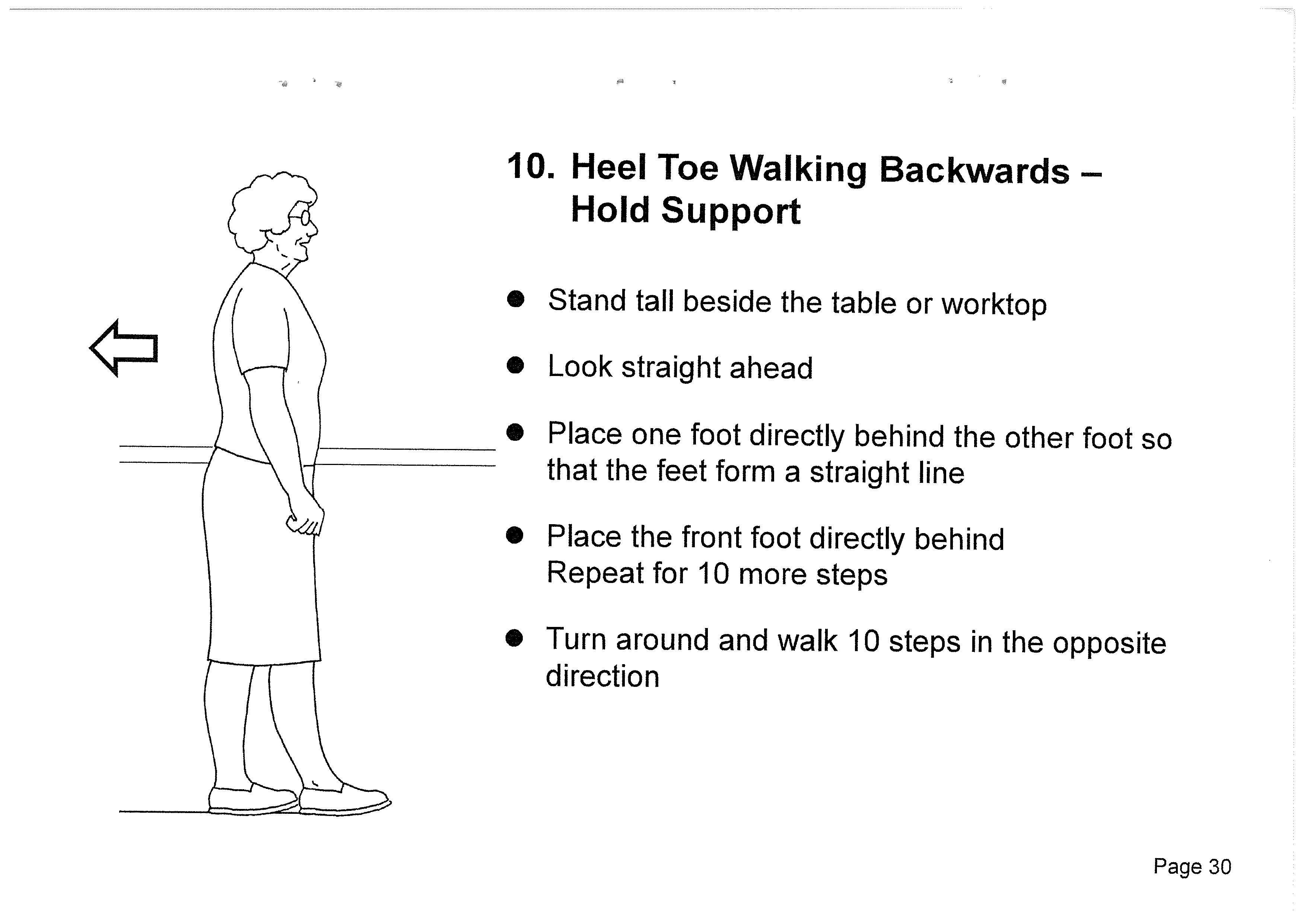 | - Heel toes walking backwards   - Stand tall beside the table or worktop - Look straight ahead - Place one foot directly behind the other foot so that the feet form a straight line - Place the front foot directly behind - Repeat for 10 more steps - Turn around and walk 10 steps in the opposite direction. | |  |  |
| 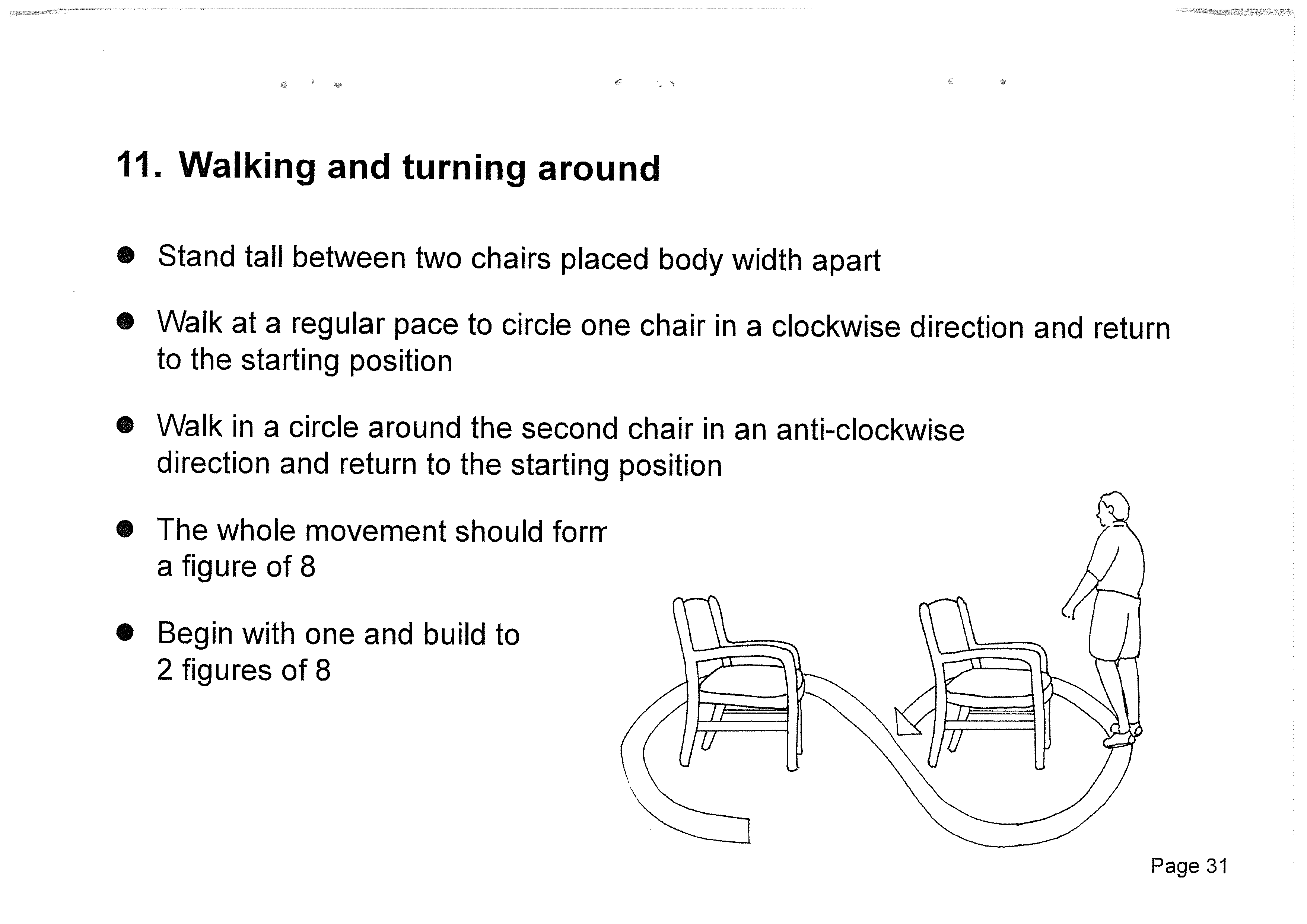 | - Walking and turning around   - Stand tall between two chairs placed body width apart - Walk at a regular pace to circle on chair in a clockwise direction and return to the starting position - Walk in a circle around the second chair in a anti-clockwise direction and return to the starting position - The whole movement should form a figure of 8 - Begin with one and build to 2 figures of 8. | |  |  |
| 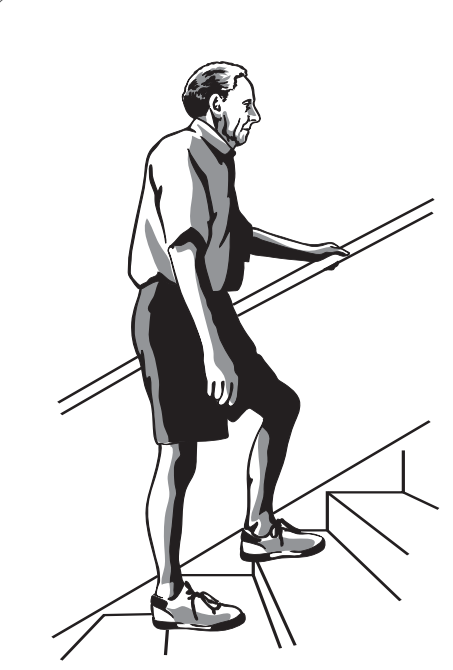 | - Stair walking as part of your exercises   - Ask your exercise instructor, physiotherapist or falls prevention practitioner if you should be doing stair walking as exercise - Hold on to the hand-rail for this exercise - Go up and down the stair for 10 times. | |  |  |
| **Cool-down session**  Cool-down exercises help you to relax and recover from exercise and maintain flexibility | | | |  |
| 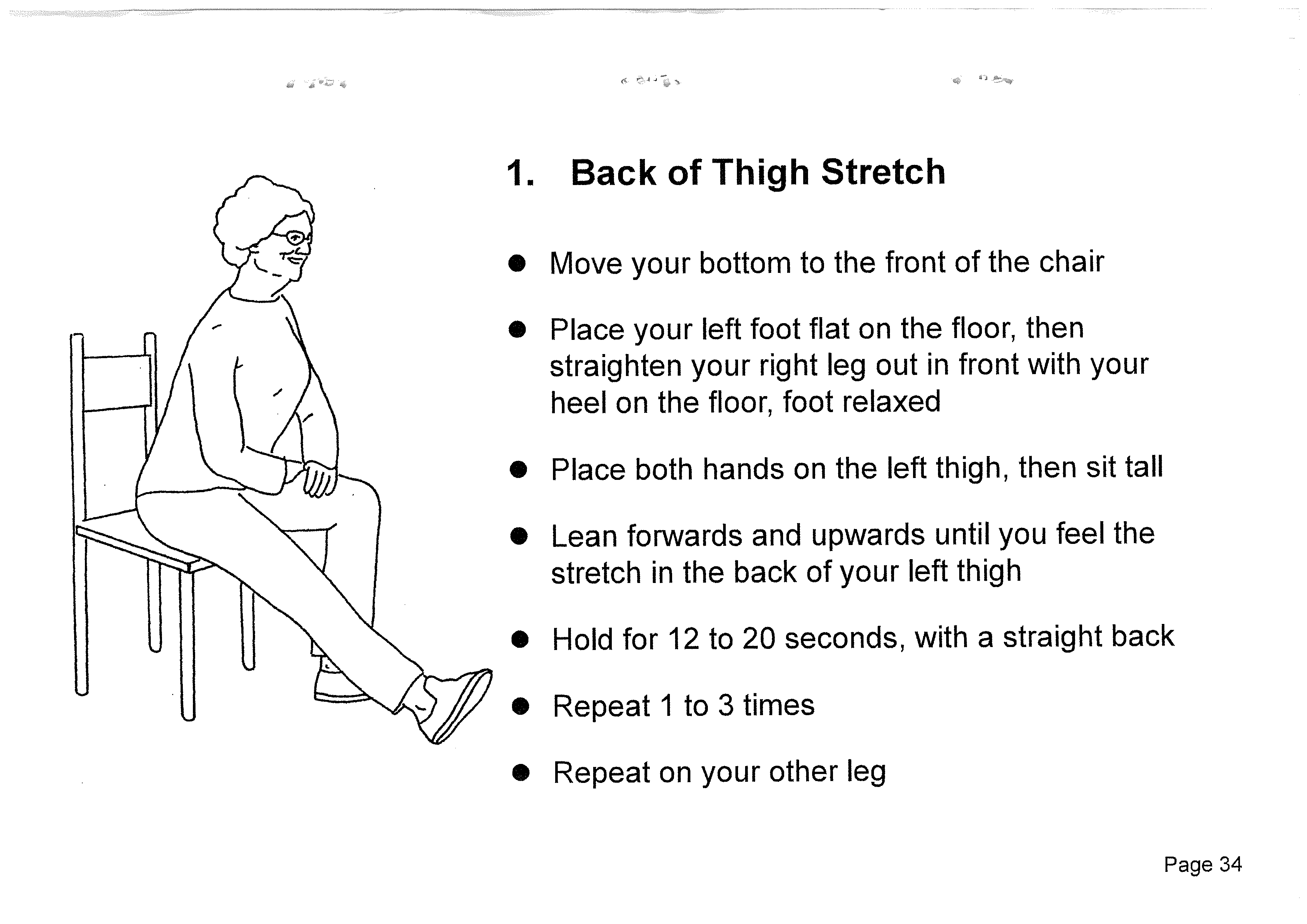 | - Back of thigh stretch   - Move your bottom to the front of the chair - Place your left foot flat on the floor, then straighten your right leg out in front with your heel on the floor, foot relaxed - Place both hands on the left thigh, then sit tall - Lean forwards and upwards until your feel the stretch in the back of your left thigh - Hold for 10 to 20 seconds, with a straight back - Repeat 1 to 3 times - Repeat to your other leg. | |  |  |
| 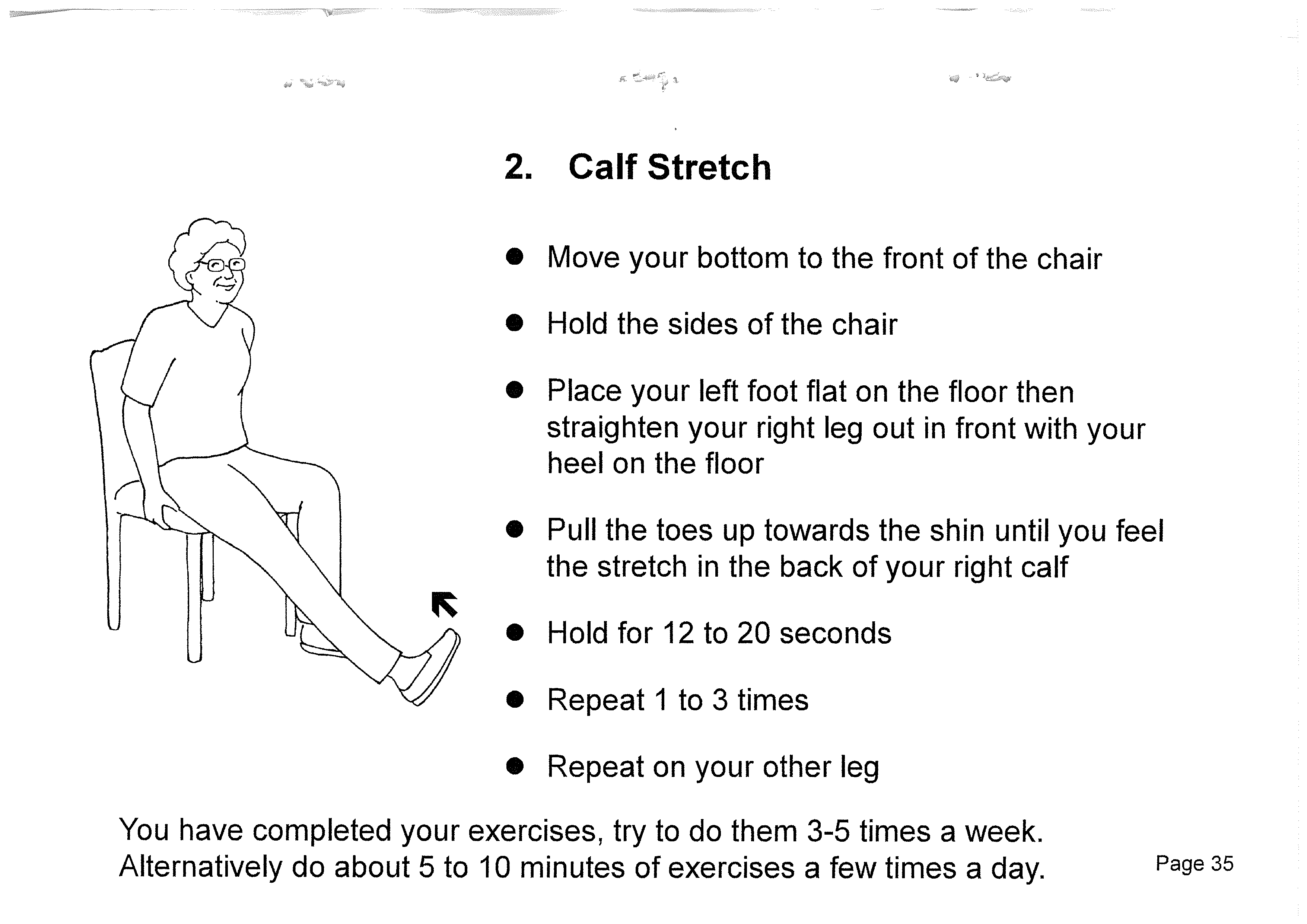 | - Calf stretches   - Move your bottom to the front of the chair - Hold the sides of the chair - Place your left foot flat on the floor then straighten your right leg out in front with your heel on the floor - Pull the toes up towards the shin until you feel the stretch in the back of your right calf - Hold for 12 to 20 seconds - Repeat 1 to 3 times - Repeat on your other leg. | |  |  |
