# Supplementary material for: Comparison of balance changes after inspiratory muscle or Otago exercise training
Source: PLoS One. 2020 Jan 24;15(1):e0227379. doi: 10.1371/journal.pone.0227379 (PMC6980667; doi:10.1371/journal.pone.0227379)
Supplement: S2 Appendix — (DOCX) [file pone.0227379.s002.docx]

| Week number | Monday | | Tuesday | | Wednesday | | | Thursday | | Friday | | | Saturday | | Sunday | | |
| --- | --- | --- | --- | --- | --- | --- | --- | --- | --- | --- | --- | --- | --- | --- | --- | --- | --- |
| Week number | Monday | | Tuesday | | Wednesday | | | Thursday | | Friday | | | Saturday | | Sunday | | |
|  | Level | Breaths | Level | Breaths | Level | | Breaths | Level | Breaths | Level | | Breaths | Level | Breaths | Level | Breaths |  |
| Morning |  |  |  |  |  |  | |  |  |  |  | |  |  |  |  |  |
| Evening |  |  |  |  |  |  | |  |  |  |  | |  |  |  |  |  |

How did you feel, and did you complete all breath without stopping?

Appendix 2 - Training diary used by the inspiratory muscle training group.

Please use the following codes to record notes on training:

**A**  Trained as expected **G** Did not train (too unwell)

**B** Trained less than expected **H** Did not train (too tired)

**C** Did not train (forgot) **I** Did not train (other reason)

**D** Did not training (too busy) **J** Increase training load

**E** Did not train (too difficult) **K** Had to stop during the 30 breaths (please indicate why)

**F** Did not train (lack of motivation) **L** Train but felt uneasy to complete the 30 breaths (please indicate why)

| Monday |  | Thursday |  |
| --- | --- | --- | --- |
| Tuesday |  | Friday |  |
| Wednesday |  | Saturday |  |
|  |  | Sunday |  |

The training diary was used to monitor adherence to inspiratory muscle training. Participants were instructed to record the number of breaths, the level of training load for each session and also comment on each training section.
