# Supplementary material for: Comparison of balance changes after inspiratory muscle or Otago exercise training
Source: PLoS One. 2020 Jan 24;15(1):e0227379. doi: 10.1371/journal.pone.0227379 (PMC6980667; doi:10.1371/journal.pone.0227379)
Supplement: Attachment — Submitted filename: Responses to reviewers 4.0.docx [file pone.0227379.s003.docx]

| **Author reply:**  We thank all the reviewers for their insightful and constructive comments, which have allowed us to enhance the quality and clarity of the manuscript. Below are our responses and clarifications, which we hope to address the queries and concerns of the reviewers. Amendments to the manuscript are highlighted in red within the manuscript text. | | | |
| --- | --- | --- | --- |
| **N** | **Reviewer Comments** | **Authors Responses** | **Action in the manuscript** |
| 1 | To enhance the reproducibility of your results, we recommend that if applicable you deposit your laboratory protocols in protocols.io, where a protocol can be assigned its own identifier (DOI) such that it can be cited independently in the future. For instructions see: http://journals.plos.org/plosone/s/submission-guidelines#loc-laboratory-protocols | Yes, we agree on the importance of transparency to enhance study reproducibility.  We have therefore added the following sentence to the manuscript.  “To enhance study reproducibility a step-by-step guide of the procedures is available at dx.doi.org/10.17504/protocols.io.8vhhw36” | Lines 102 - 104 |
| 2 | The article is well written however there are serious concerns with blinding and objectivity considering the PI of the study conducted the assessments and administered the intervention. Why this is not a fatal flaw to the design will need to be described and highlighted in the limitations section.  Statistical analyses and their interpretation’s seem generally appropriate. These findings provide the first evidence that IMT improves balance and physical performance for healthy older adults compared to Otago exercise training. | We appreciate the valid concern. Due to the experimental nature of this study and funding constraints (it was a PhD project), we were unable to employ blinded assessors and instructors.  We were aware of this in planning, and therefore ensured that the principal investigator was competency-trained in the assessment methods (particularly in validating laboratory testing methods to be used ‘in the field’) and in delivering the Otago exercise programme (trained by nursing staff at The Royal Bournemouth and Christchurch Hospital NHS Foundation Trust)  The authors confirm that the interventions were delivered accordingly to hospital guidelines, as both groups showed improvements in balance (mini-BEST) in accordance with previous research.    Nevertheless, the limitation has been considered, made more explicit and the following section as been added to the Limitations paragraph (Lines 486 – 497):  “To our knowledge, this is the first study to compare an established physical training intervention, to a novel respiratory training intervention which may influence mobility and dynamic balance. We were limited in delivering the IMT in care home settings, due to heterogeneity in the health status of our population (e.g. a large proportion of residents have COPD and respiratory conditions).  We were also unable to employ blinded assessors or instructors, due to funding constraints to compare IMT against the OEP, in terms of improving participant outcomes in balance and physical performance. However, the principal investigator (FF) received competency training in physical training, respiratory muscle training, and technical aspects of all experimental equipment and underwent training at The Royal Bournemouth and Christchurch Hospital NHS Foundation Trust.“ | Lines 486-497 |
| 3 | Abstract - Please be more specific in introduction of the abstract for the term “community dwellers”. Are these older adults with balance impairment? | The authors observed the effects of IMT and OEP on balance and physical performance outcomes with healthy participants. The term ‘community-dwellers’ relates to older adults who are independent in their home and community environments, and who do not rely on assistive or daycare, nor on family or friends. They had no balance impairments at the time of testing ( Steinmetz, H.M. and Hobson, S.J., 1995).  To help the readers the term “healthy” has been now introduced in the abstract. | Page 2 |
| 4 | Introduction: The introduction describes the OEP as a successful approach to improve TUG however this study did replicate that finding. This should be discussed. | These results were unexpected and likely attributable to the wide age range (and relatively low statistical power, as was calculated for the primary outcome: balance).  Also, it is worth to consider that all the participants involved in this study were healthy older adults with a low risk of fall. We believe that a frailer population (i.e. older adults that had experienced a fall in the past three months ) would have shown a significant improvement in balance compare to what we reported.  The following sentence has been modified in the discussion:  “the unpredicted absence of significant improvements in TUG performances for the OEP group is attributable to the wide age range of the participants (75 to 89 years) and therefore, to the heterogeneity of their mobility.” | Lines 441 - 443 |
| 5 | Methods: Participant characteristics - is it not chronic obstructive pulmonary disease?  How was it determined that patients had chronic lung disease? Self-report? If so, what about those that might have undiagnosed disease? | No, we determined whether patients had ‘chronic lung disease’ (or other respiratory disease) in initial screening.  Firstly, we asked the participants whether they were diagnosed with any respiratory conditions (including asthma and COPD) for a subjective opinion.  Secondly, with the first respiratory measurement (i.e. spirometry test, in particular forced vital capacity and forced expiratory volume in 1 second) it was possible to objectively understand whether or not there were signs of obstructive pulmonary disease.  This is made possible by the Tiffeneau-Pinelli index, as the equipment was able to produce in a real-time report immediately after the test had been undertaken.  This clinical test would identify any individuals at risk of or having undiagnosed disease.  Further details about spirometry measurements and Tiffeneau-Pinelli has been included in the protocol.io dx.doi.org/10.17504/protocols.io.8vhhw36 | Page 5 |
| 6 | It is stated that “staff” were recruited to perform OEP. Were they required to have certifications and/or previous training?  Were they trained to specifically perform OEP? It also stated that the “classes were delivered by the PI” but no details on training were given. This is of particular importance considering the OEP in this study does not match previous reports of improvements. | Staff members of the care homes did not deliver the OEP.  “The staff members of the care homes were recruited in an assistive role, to organise the facilities in which the OEP was delivered and to remind their guests about the appointments.“  The Principal Investigator (FF) delivered the OEP, as they received specialist training from the Geriatric Medicine Department, The Royal Bournemouth and Christchurch Hospital NHS Foundation Trust  To report the training progression and complete training regime used, a copy of the intervention has been added as an appendix to the document, (available for publication as a supplement). | Line 86 - 88 |
| 7 | Seems alcohol should have been suggested to be avoided more than 2 hours before the exercise. | Yes, it was advised not to consume any substance that is known to alter balance ability prior to testing.  To clarify, “more than” has been added to the document | Line 95 |
| 8 | It is stated that the PI performed assessments. Because he/she delivered the intervention there are major concerns about objectivity. | As reported in comments N 2, the assessments used to measure balance and physical performance are by definition objective measures, and the error range in each measurement was found to be in within tests reliability.  Additionally, the PI has experience in delivering training interventions and with the used assessments. Due to the project funding and technical support, it would not have been possible to train the care staff to assess and/or instruct, nor would it to train a researcher. The PI and research team were cognisant of this limitation and designed the protocol to limit bias.  To clarify these queries we have added details to the Limitations paragraph (please refer to point 2). | Lines 502 - 513 |
| 9 | More details on progression of the interventions is needed as well as added details on compliance to the interventions | To enhance the following section, a copy of the Otago exercises as adopted by the Geriatric Medicine Department, NHS Christchurch Hospital, has been added in appendixes. This will allow the reader to gain insight into our specific programme and how we standardised progression. Due to manuscript word limits and the paper’s overall focus, it is no possible to include this detail in the manuscript. Also, for the same reasons, a copy of the IMT training diary has been included in appendixes for the reader.  Please refer to line 226 for details on training interventions:  “The number of repetitions and the level of resistance were tailored to participants` physical fitness and progressively increased (by 0.5 kg if they were able to increase the number of repetitions by two). The resistance of each exercise was increased through ankle weights (ranging from 0.5 - 5 kg). Exercise sessions were conducted between 10:00 and 12:00. Participants were also instructed to walk for a minimum of 30 minutes at least twice a week (self-report), on the days in which the OEP was not performed. Attendance at the exercises classes was recorded by the principal investigator (FVF); participants were also instructed to cease exercising if they experienced any physical or mental discomfort.”  From line 358 for details about training diaries:  “Adherence to IMT was self-reported via training diaries, in which participants were instructed to record the number of breaths and the level on training load for each session (Appendix 2).” | Lines 224-233 |
| 10 | The results and discussion highlight “(12/19 and 14/18)” but it is unclear what these numbers are referring to. | The numbers referred to the participants that completed the interventions:  “12 participants out of 19 successfully completed IMT. 14 participants out of 18 successfully completed OEP”.  These parentheses have been now removed as the values are shown in percentage, and the CONSORT flow chart also illustrates training compliance.  These values are also documented on line 352 as:  “63% ~~(12/19 sessions)~~ for IMT and 78% ~~(14/18 sessions)~~ for OEP” | Line 255 |
| 11 | The hypothesized mechanisms for noted improvements should be discussed/expanded. | The hypothesised mechanisms behind the observed improvements in inspiratory muscle strength and balance were not the aim of this study, and in explaining in detail, we run the risk of a speculative discussion. Yet, we agree that it is important to discuss the potential physiological mechanisms causing these improvements. For clarity we have now included the following:  ***Potential mechanism 1 - upper-body and lower-body segmental linkage*** (line 403 – 411)  ***Potential mechanism 2 - intra-abdominal pressure*** (line 413 – 420).  The two sub-sections are followed by recommendations for further research to investigate the relationship between inspiratory muscle and balance (line 421 – 427)  “The authors believe that to understand the potential relationship between inspiratory muscle activation and balance, other measurements, such as EMG, or intra-abdominal (gastric) balloon catheter, are necessary. Further research is required to investigate both i) the efficacy of IMT as a falls prevention intervention in frailer populations, and ii) the potential physiological adaptations conferred from an intervention tailored to improve inspiratory muscles strength and balance ability.” | Lines 395-427 |
| 12 | While revising your submission, please upload your figure files to the Preflight Analysis and Conversion Engine (PACE) digital diagnostic tool, https://pacev2.apexcovantage.com/. PACE helps ensure that figures meet PLOS requirements. To use PACE, you must first register as a user. Registration is free. Then, login and navigate to the UPLOAD tab, where you will find detailed instructions on how to use the tool. If you encounter any issues or have any questions when using PACE, please email us at figures@plos.org. Please note that Supporting Information files do not need this step. | Figures have been revised with Preflight Analysis and Conversion Engine (PACE) digital diagnostic tool. |  |
|  | We note that you have a patent relating to material pertinent to this article. Please provide an amended statement of Competing Interests to declare this patent (with details including name and number), along with any other relevant declarations relating to employment, consultancy, patents, products in development or modified products etc. Please confirm that this does not alter your adherence to all PLOS ONE policies on sharing data and materials, as detailed online in our guide for authors http://journals.plos.org/plosone/s/competing-interests by including the following statement: "This does not alter our adherence to PLOS ONE policies on sharing data and materials.” If there are restrictions on sharing of data and/or materials, please state these. Please note that we cannot proceed with consideration of your article until this information has been declared. | That is correct, as stated in the ‘Declaration of Interests’, a co-author (AM) is named on the following patents (European Patent Office Patent No. EP2303417B1 - Dynamic inspiratory muscle training device. United States Patent No. 8 459 255 - Dynamic inspiratory muscle training device  Additional examples of international registration are accessible via <https://patents.google.com/patent/US8459255B2/en>  However, AM has no current or future financial interest in either of these patent.  AM is also the author of the following books on inspiratory muscle training (Respiratory Muscle Training: Theory and Practice. *Elsevier Health Sciences, 2013.* Breathe strong, perform better. *United States of America: Human Kinetics, 2011).*  We have now uploaded an amended statement of Competing Interests to declare this patient (line 532 – 541)  We can confirm that this does not alter our adherence to any one of the PLOS ONE policies on data and materials sharing. | Page 26  Lines 536 - 544 |
